# The Development of Spatial Memory Analyzed by Means of Ecological Walking Task

**DOI:** 10.3389/fpsyg.2019.00728

**Published:** 2019-03-29

**Authors:** Pierpaolo Sorrentino, Anna Lardone, Matteo Pesoli, Marianna Liparoti, Simone Montuori, Giuseppe Curcio, Giuseppe Sorrentino, Laura Mandolesi, Francesca Foti

**Affiliations:** ^1^ Department of Engineering, Università degli Studi di Napoli Parthenope, Naples, Italy; ^2^ Department of Movement Sciences and Wellbeing, Università degli Studi di Napoli Parthenope, Naples, Italy; ^3^ Department of Biotechnological and Applied Clinical Sciences, University of L’Aquila, L’Aquila, Italy; ^4^ Istituto di Diagnosi e Cura Hermitage Capodimonte, Naples, Italy; ^5^ Institute of Applied Sciences and Intelligent Systems, CNR, Naples, Italy; ^6^ Department of Humanistic Studies, University Federico II, Naples, Italy; ^7^ Department of Medical and Surgical Sciences, Università degli studi Magna Græcia di Catanzaro, Catanzaro, Italy

**Keywords:** spatial exploration, cognitive map, spatial memory, behavioral task, children

## Abstract

The present study is aimed at investigating the development of spatial memory in pre-school children aged 4–6 years using an ecological walking task with multiple rewards. The participants were to explore an open space in order to find nine rewards placed in buckets arranged in three spatial configurations: a Cross, a 3 × 3 Matrix, and a Cluster composed of three groups of three buckets each. Clear age-related improvements were evident in all the parameters analyzed. In fact, there was a general trend for younger children to display worse performance than the older ones. Moreover, males performed better than females in both the search efficiency and visiting all buckets. Additionally, the search efficiency proved to be a function of the difficulty of the configuration to be explored: the Matrix and Cluster configurations were easier to explore than the Cross configuration. Taken altogether, the present findings suggest that there is a general improvement in the spatial memory abilities in preschoolers and that solving an open space task could be influenced by gender. Moreover, it can be proposed that both the procedural competences and the memory load requested to explore a specific environment are determined by its specific features.

## Introduction

Navigational abilities are strongly correlated with spatial memory processes, including both procedural and declarative components. In fact, when encoding the spatial relationships of an environment (declarative spatial knowledge), one has to learn “how” to move in that environment (procedural spatial knowledge), thus suggesting that procedural competences and mapping abilities are equally necessary for efficient exploration (O’Keefe and Nadel, 1978; Mandolesi et al., 2009). An important role in these processes is played by spatial working memory, which is involved in retention and processing of visuospatial information (Baddeley, 1986; Fenner et al., 2000) and correlated with attentional control (Awh et al., 2006; Gigliotta et al., 2017). In fact, when exploring a new environment, besides the awareness of spatial features, one also needs to temporarily store and manipulate visuospatial information in order to find objects or reach a target, thus inhibiting distracting stimuli (Flouri et al., 2018).

Although spatial competences appear very early and are age-related (Acredolo, 1977; Hermer and Spelke, 1994; Lehnung et al., 1998; Nardini et al., 2009; Bullens et al., 2010; Piccardi et al., 2014), these cognitive processes are not fully developed in children younger than about 7 years of age, and mapping abilities only appear at 10 years of age (Overman et al., 1996; Lehnung et al., 1998; Mandolesi et al., 2009). Behavioral studies in this field are in accordance with neuroimaging research showing functional maturation of cerebral correlates of spatial competences in late childhood and adolescence (Klingberg, 2006). Recently, it has been evidenced that spatial working memory develops throughout childhood and is associated with the maturation of specific white matter tracts (Krogsrud et al., 2018). These findings are in accordance with a recent fMRI study investigating the neurological mechanisms underlying the ability to orient oneself in a virtual environment. In fact, children from 8 to 10 years of age displayed increased neural activity in cerebral areas associated with visuospatial processing and navigation, such as the left cuneus and the mid-occipital area, the left inferior parietal region and precuneus, the right inferior parietal cortex, the right precentral gyrus, the cerebellar vermis, and the medial cerebellar lobes bilaterally (Murias et al., 2019).

In developmental research, it has been seen that, at around 6 months of age, infants possess the ability to use visual landmarks (Acredolo and Evans, 1980; Crowther et al., 2000; Lew et al., 2000) and, by the end of the first year, they are aware of their own position in the environment and learn information about the spatial context in which they are located (through movement and proprioceptive information) (Loomis et al., 1993). In this context, it has been evidenced that 5-year-old children are able to find locations in a spatial array, starting from a novel perspective, using landmarks alone (Nardini et al., 2006).

The evidence regarding gender differences in the development of spatial abilities is more controversial. On one hand, it is clear that, from puberty onward, males display a more efficient use of spatial competencies than females, which might be related to the maturation of specific cerebral structures such as the corpus callosum, the hippocampus, and the frontal cortex (Giedd et al., 1999; Vuontela et al., 2003; Alejandre-Gomez et al., 2007; Méndez-López et al., 2009). On the other hand, the evidence of gender differences during childhood is more debated. Some behavioral studies evidenced that males and females use different strategies to explore the environment and to acquire spatial information (Lawton, 1994, 1996; Robinson et al., 1996; Astur et al., 1998, 2004; Sandstrom et al., 1998; Gibbs and Wilson, 1999; Beilstein and Wilson, 2000; Grön et al., 2000; Blanch et al., 2004). Recently, it has been observed that in some spatial competencies, as well as in object localization, females perform better than males do before the age of 13 (Bocchi et al., 2018).

However, further evidence documented similar performances in both genders with regard to spatial tasks (Linn and Petersen, 1985; Aliotti and Rajabiun, 1991; Anderson and Lajoie, 1996; Overman et al., 1996; Lehnung et al., 1998, 2003; Nichelli et al., 2001; Leplow et al., 2003).

In a previous work, we analyzed the spatial abilities of preschoolers and schoolers using a large-scale radial arm maze (RAM), an ecological instrument that allows the analyses of different facets of spatial function (Mandolesi et al., 2009). In particular, the RAM consists of a central area from which a number of identical arms radiate. At the end of each arm, there is a hidden reward. In the free-choice paradigm, the subject is required to recover all the rewards without making mistakes. Provided that there is only one reward per arm, and that revisiting an arm is considered a mistake, the subject will need both declarative and procedural competencies to perform the task. In this specific setting, we showed a clear age- and gender-related effect in all the parameters analyzed (Mandolesi et al., 2009). In short, younger children (3.5–4 years) performed poorly as compared to older ones (4 years older), and females exhibited acquisition of spatial competences earlier in comparison to males up to 5.5 years old (Mandolesi et al., 2009). However, in the RAM task, children have to find the hidden rewards according to a fixed spatial configuration, and the searching strategies are limited by the number of alternative routes. To overcome this limitation, we investigated the spatial abilities of children aged 4–6 years in a large-scale task without any spatial constraint, so as to make the task harder and potentially uncover developmental trends of spatial memory in this age range, as well as possible gender differences and specific environmental features that might facilitate the exploration. In this spatial task, the child is free to move, adopting exploratory behaviors in accordance with the environment. Thus, the environmental affordances influence the construction of the search strategies as well as the knowledge of the positions of the rewards (Foti et al., 2011, 2015). In particular, in the present study, the participants were asked to explore an open space to search for nine rewards hidden in buckets arranged in three spatial configurations: a Cross, a 3 × 3 Matrix, and a Cluster composed of three groups of three buckets each. We believe that the analysis of spatial exploration in open environments, without any constraints, could increase our knowledge of the development of spatial abilities in children. In the current study, we hypothesize that the characteristics of the environment define the specific spatial memory competencies needed to explore it and, consequently, the implementation of appropriate navigational strategies. For this reason, we expect that the difficulties in exploring will decay as a function of age. Furthermore, we went on to evaluate the locomotion of the participants. To do this, we computed the total distance travelled to complete a task. This information is relevant as it has been shown that locomotion facilitates the acquisition of spatial competencies (Lehnung et al., 2003).

## Materials and Methods

### Participants

Thirty-six healthy Italian children (17 M and 19 F) aged from 4 years and 1 month (4.1) to 6 years and 2 months (6.2) (mean age: 5.3 ± SEM 1.3) participated in the present study. Participants were divided into two groups based on the classes of the kindergarten: group I (*N* = 18; 9 M and 9°F; mean age: 4.7 ± 0.8) and group II (*N* = 18; 10 M and 8°F; mean age: 5.8 ± 0.9). All the children attended a public kindergarten in Southern Italy, and none had had previous experience with the multiple reward task. Moreover, none of the children presented neurological or neuropsychological disorders, and all had normal or corrected-to-normal vision. To verify typical cognitive development, all participants were assessed by Raven matrices test (Raven, 1938; Raven Court and Raven, 1995). Written informed consent to perform the task was obtained from the children’s parents. The study was conducted according to the 1964 Declaration of Helsinki and was approved by the Internal Review Board of the University of L’Aquila.

### Apparatus

The apparatus was situated in open-air, in a large garden, and consisted of nine orange plastic buckets (18 cm wide × 28 cm high) containing the reward (a little-colored ball). The buckets, along with a swinging cover, were arranged in three different spatial configurations as described in the *Procedures* section. The apparatus was surrounded by extra-maze cues (trees, swings, benches, etc.) held in constant spatial relations among each other throughout the experiment. During the test phases only, children could see or have physical access to the three different spatial configurations. In order to increase the motivation to pick up the rewards, at the end of each trial, the child received a reward (a little toy) in exchange for all the colored balls found in the buckets (Foti et al., 2011, 2015).

### Procedures

**Figure 1:**
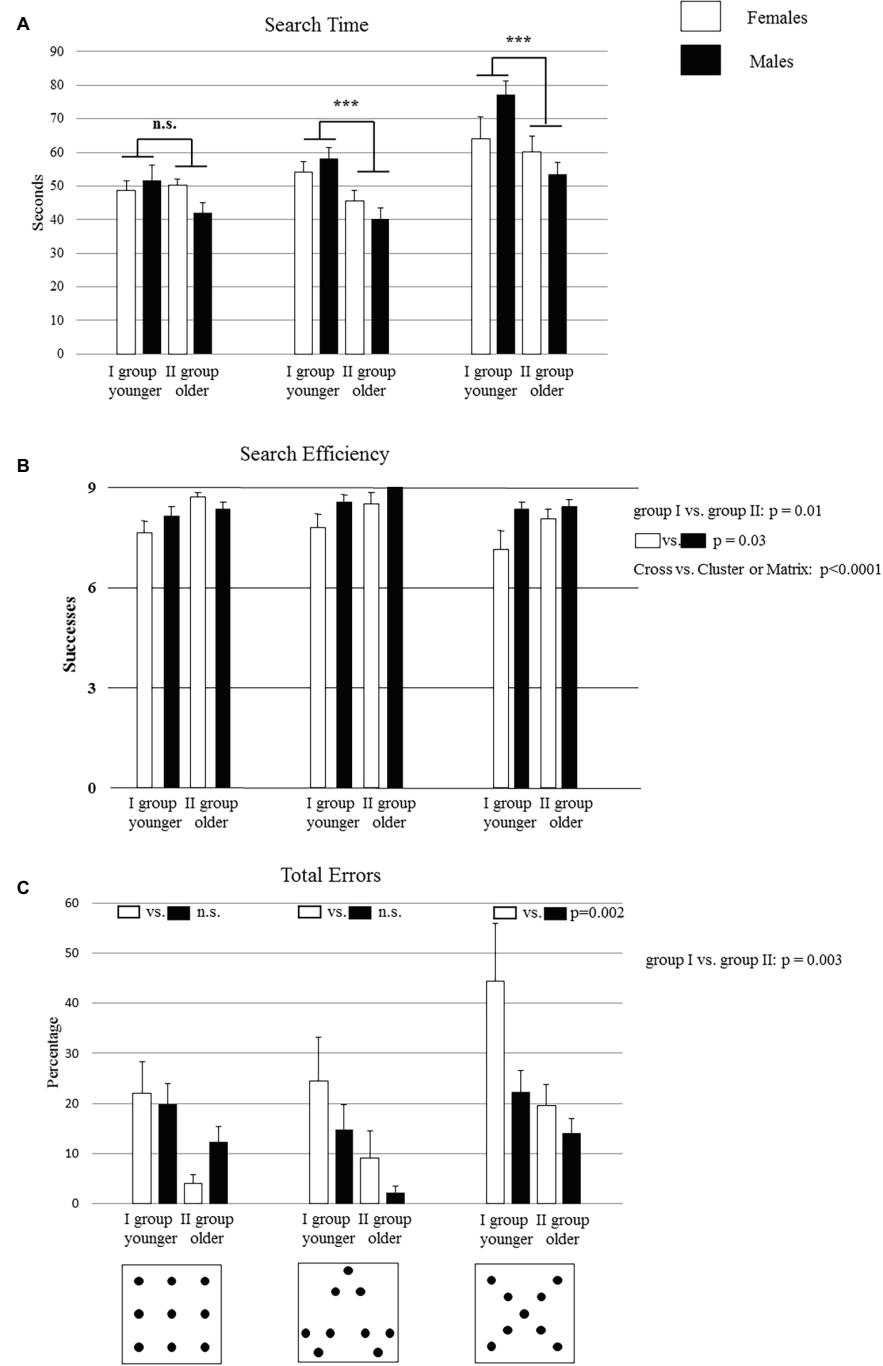
Performances of group I and group II on the search task in Matrix, Cluster, and Cross configurations. Bucket arrangement in the three configurations is depicted in the figures below the graphs **(A,B,C)**. Data are presented as mean ± SEM. Asterisks and the *p* values inside the graphs **(A,C)** indicate the significance level of *post hoc* comparisons on the second-order interactions: ***p < 0.0005. The *p* values of the main factors are reported on the right side of each graph.

**Figure 2:**
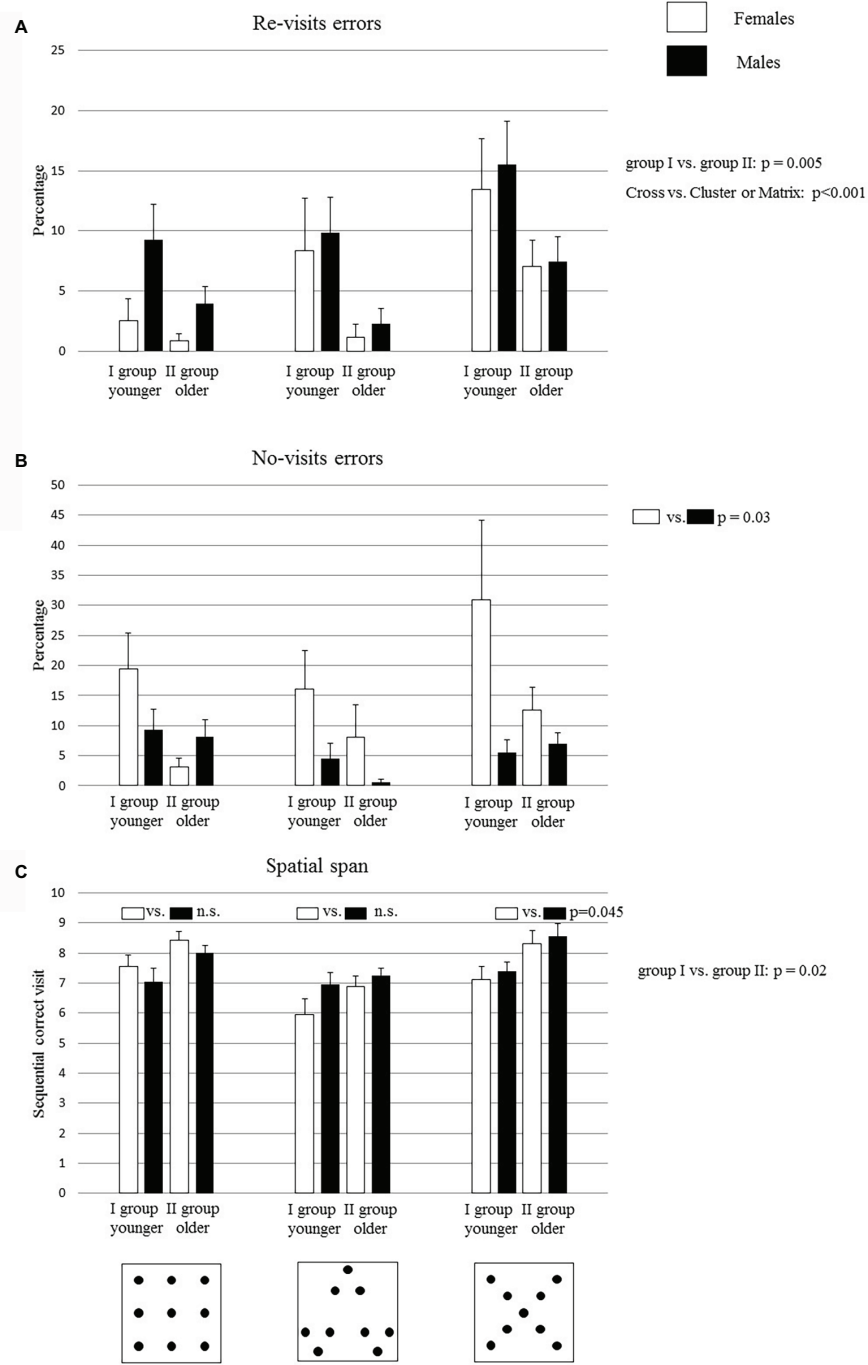
Performances of group I and group II on the search task in Matrix, Cluster, and Cross configurations. Bucket arrangement in the three configurations is depicted in the figures below the graphs. Data are presented as mean ± SEM **(A,B,C)**. The *p* values inside the graph **(C)** indicate the significance level of *post hoc* comparisons on the second-order interaction. The *p* values of the main factors are reported on the right side of each graph.

Spatial configurations were derived from previous experimental studies that demonstrated reliability in emphasizing task features and have been accurately described in our previous research (Foti et al., 2011, 2015). In the Matrix configuration, the buckets were arranged 4 m apart in a 3 × 3 square matrix. In the Cross configuration, the buckets were arranged 4 m apart in an “X” formation. In the Cluster configuration, the buckets were arranged 4 m apart, in triplets 120° away from each other (in the lower part of Figures 1, 2, the arrangement of the buckets in the three configurations is depicted).

Each child was allowed to freely explore the apparatus to retrieve the rewards. A trial ended when all nine rewards had been collected or 30 visits (correct or wrong) had been made. Since the buckets were never filled with two rewards in the same trial, the optimal performance consisted of visiting each bucket only once, collecting nine rewards through nine visits. A bucket was considered visited when the child looked inside the bucket. An error was recorded when the child re-visited a bucket already visited during the same trial or when a bucket was never visited. Each participant performed two trials a day (inter-trial interval: 2 h) with a given spatial configuration. On the first day, the children performed two trials with one spatial configuration. The next day, they performed two trials with a different spatial configuration. On the third day, they performed two trials with the remaining spatial configuration. The order of presentation of the three configurations was randomized among children.

At the beginning of the first test day, the experimenter used the same simple verbal instructions to explain the task to each child (“The game is to find some little colored balls. Do you see the orange buckets? You have to reach a bucket, take the little ball inside, until you have collected all the balls. Go and have fun!”). No other instruction or verbal encouragement was provided during the testing. Each participant wore an actigraph device (wActiSleep-BT, ActiGraph, Pensacola, Florida) to record the steps taken during the exploration of each configuration.

### Behavioral Parameters

In each of the two trials of a given configuration, the following parameters were analyzed: the s*earch time,* i.e., the time (in seconds) to complete the task; the s*earch efficiency*, i.e., the number of appropriate visits (successes) performed in the trial; the *total errors,* i.e., the percentage of total errors out of the total visits (considering both re-visits (visiting a previously depleted bucket) and no-visits to a bucket (skipping a bucket)); and the *re-visit errors,* the *no-visit errors*, and the *spatial span*, i.e., the longest sequence of correct visits. Moreover, in order to evaluate the locomotion, we calculated the *total distance* (in centimeters) traveled to complete the task.

### Drawings

In order to evaluate the graphical and mental representation mapping abilities, after the second trial of each configuration, all children were asked to draw the setting where they had just “played.” Thus, each child drew three drawings, one for each configuration. No instructions were provided either about representing the individual objects, the global setting, or about indicating how many buckets (or rewards) were present in the setting.

In examining the drawings of the three spatial configurations, we evaluated the type of representation, an index rating the egocentric/allocentric ratio of drawings, using a 5-point Likert scale (from 1: clearly egocentric, to 5: clearly allocentric), according to Foti et al., 2018. To objectively assess this parameter, we asked two coders, blind to experimental conditions and expert in mental spatial representations and human navigation, to score each drawing according to its egocentricity/allocentricity. The scoring was considered reliable only when the Cohen’s kappa coefficient showed sufficient consistency (*k* > 0.75).

### Statistical Analysis

The results of each participant belonging to experimental groups were presented as mean values of the two trials of any configuration ± SEM. The data were first tested for normality (Shapiro-Wilk normality test) and homoscedasticity (Levene test) and then compared using three-way analyses of variance (ANOVA) by applying the mixed model for the independent variables (Age and Gender) and repeated measures (Configurations), followed by *post hoc* using Duncan’s test.

Since in the present study a number of analyses were run, controlling for the alpha inflation was needed. We controlled the proportion of type I errors among all rejected null hypotheses by setting the false discovery rate (FDR) to 0.05. The FDR was estimated through the procedure described in Storey and Tibshirani, 2003. In our results, the 0.05 level of significance corresponded to an FDR < 0.05.

## Results

### Search Time

With regard to the time spent to complete the test, a three-way ANOVA (Age × Gender × Configuration) was used. Results are reported as *F* statistic (*F*), statistical significance (*p*), and bias effect size estimation (${\eta_{p}}^{2}$). The statistical analysis revealed significant Age (*F*_1,32_ = 11.71, *p* = 0.002, ${\eta_{p}}^{2}$ = 0.27) and Configuration (*F*_2,64_ = 36.99, *p* < 0.000001, ${\eta_{p}}^{2}$ = 0.53) effects, while the Gender (*F*_1,32_ = 0.0001, *p* = 0.99) effect was not significant. Also, the first-order Age × Gender (*F*_1,32_ = 4.9, *p* = 0.03, ${\eta_{p}}^{2}$ = 0.13) and Age × Configuration (*F*_2,64_ = 3.78, *p* = 0.03, ${\eta_{p}}^{2}$ = 0.10) interactions were significant. Conversely, the first-order Gender × Configuration (*F*_2,64_ = 1.08, *p* = 0.34) and the second-order Age × Gender × Configuration (*F*_2,64_ = 1, *p* = 0.37) interactions were not significant.

As revealed by the *post hoc* comparisons performed on the first-order Age × Gender interaction, the male children of group II were significantly faster than the male children of group I (*p* = 0.001), while the two groups of females took similar times (*p* = 0.4). Moreover, the *post hoc* comparisons performed on the first-order Age × Configuration interaction showed that, in the Matrix configuration, group I took a similar time in comparison to group II (*p* = 0.15). However, in the Cross and Cluster configurations, group I was significantly slower than group II (at least *p* = 0.0005) (Figure 1A).

### Search Efficiency

A three-way ANOVA (Age × Gender × Configuration) showed significant Age (*F*_1,32_ = 6.94, *p* = 0.01, ${\eta_{p}}^{2}$ = 0.18), Gender (*F*_1,32_ = 4.99, *p* = 0.03, ${\eta_{p}}^{2}$ = 0.13), and Configuration (*F*_2,64_ = 3.69, *p* = 0.03, ${\eta_{p}}^{2}$ = 0.10) effects. None of the interactions were significant (Age × Gender: *F*_1,32_ = 2.12, *p* = 0.15; Age × Configuration: *F*_2,64_ = 0.09, *p* = 0.91; Gender × Configuration: *F*_2,64_ = 2.57, *p* = 0.08; Age × Gender × Configuration: *F*_2,64_ = 0.47, *p* = 0.62).

Interestingly, *post hoc* comparison performed on the Age and Gender effects revealed that group II obtained higher values of search efficiency than group I (*p* = 0.01) and that male children performed better than female children (*p* = 0.03). Moreover, *post hoc* comparisons performed on the Configuration effect revealed that the Cross configuration was more difficult than the Matrix and Cluster configurations (Cross vs. Cluster or Matrix: at least *p* < 0.0001) (Figure 1B).

### Total Errors

A three-way ANOVA (Age × Gender × Configuration) revealed significant Age (*F*_1,32_ = 10.66, *p* = 0.003, ${\eta_{p}}^{2}$ = 0.25) and Configuration (*F*_2,64_ = 10.32, *p* = 0.0001, ${\eta_{p}}^{2}$ = 0.24) effects, while the Gender (*F*_1,32_ = 2.06, *p* = 0.16) effect was not significant. Moreover, also the first-order interaction Gender × Configuration was significant (*F*_2,64_ = 4.24, *p* = 0.02, ${\eta_{p}}^{2}$ = 0.12). The remaining interactions were not significant (Age × Gender: *F*_1,32_ = 1.27, *p* = 0.26; Age × Configuration: *F*_2,64_ = 0.21, *p* = 0.81; Age × Gender × Configuration: *F*_2,64_ = 0.70, *p* = 0.5). *Post hoc* comparisons performed on the Age effect revealed that group I had significantly higher total errors than group II (*p* = 0.003). Moreover, as revealed by the *post hoc* comparisons performed on the first-order Gender × Configuration interaction, the performance of female children was worse in the Cross configuration than the performance of male group (*p* = 0.002), while there were no significant differences between female and male children in the Matrix (*p* = 0.46) and Cluster (*p* = 0.07) configurations (Figure 1C).

### Re-visit Errors

A three-way ANOVA (Age × Gender × Configuration) revealed significant Age (*F*_1,32_ = 9.28, *p* = 0.005, ${\eta_{p}}^{2}$ = 0.22) and Configuration effects (*F*_2,64_ = 10.89, *p* = 0.00008, ${\eta_{p}}^{2}$ = 0.25), while Gender effect was not significant (*F*_1,32_ = 1.56, *p* = 0.22). None of the interactions were significant (Age × Gender: *F*_1,32_ = 0.22, *p* = 0.64; Age × Configuration: *F*_2,64_ = 1.04, *p* = 0.36; Gender × Configuration: *F*_2,64_ = 0.95, *p* = 0.39; Age × Gender  × Configuration: *F*_2,64_ = 0.14, *p* = 0.86). *Post hoc* comparisons on the Age effect revealed that group I had a significantly higher percentage of re-visit errors than group II (*p* = 0.005). Moreover, *post hoc* comparisons performed on the Configuration effect revealed that Cross configuration was more difficult than Matrix and Cluster configurations (Cross vs. Cluster or Matrix: at least *p* < 0.001) (Figure 2A).

### No-Visit Errors

A three-way ANOVA (Age × Gender × Configuration) revealed a significant Gender effect (*F*_1,32_ = 4.89, *p* = 0.03, ${\eta_{p}}^{2}$ = 0.13), while Age (*F*_1,32_ = 3.46, *p* = 0.07) and Configuration (*F*_2,64_ = 3.1, *p* = 0.06) effects were not significant. None of the interactions were significant (Age × Gender: *F*_1,32_ = 2.5, *p* = 0.12; Age × Configuration: *F*_2,64_ = 0.16, *p* = 0.85; Gender × Configuration: *F*_2,64_ = 2.85 *p* = 0.06; Age × Gender × Configuration: *F*_2,64_ = 1.11, *p* = 0.34). *Post hoc* comparisons performed on the Gender effect revealed that female children made more no-visit errors than male children did (Figure 2B) (*p* = 0.03).

### Spatial Span

The spatial span is represented by the longest sequence of correct visits. A three-way ANOVA (Age × Gender × Configuration) revealed significant Age (*F*_1,32_ = 11.64, *p* = 0.002, ${\eta_{p}}^{2}$ = 0.27) and Configuration (*F*_2,64_ = 12.85, *p* = 0.00002, ${\eta_{p}}^{2}$ = 0.29) effects, while Gender (*F*_1,32_ = 0.37, *p* = 0.55) effect was not significant. Moreover, the first-order interaction Gender × Configuration was significant (*F*_2,64_ = 2.94, *p* = 0.04, ${\eta_{p}}^{2}$ = 0.02), while the remaining interactions were not significant (Age × Gender: *F*_1,32_ = 0.13, *p* = 0.72; Age × Configuration: *F*_2,64_ = 0.71, *p* = 0.5; Age × Gender × Configuration: *F*_2,64_ = 0.32, *p* = 0.73).

*Post hoc* comparisons performed on the Age effect showed that group II exhibited higher values of span than group I (*p* = 0.002). Moreover, *post hoc* comparisons performed on the first-order interaction Gender × Configuration showed that male children exhibited significantly higher values of span than female children in the Cross configuration (*p* = 0.045), while there were no significant differences between female and male children in the Matrix (*p* = 0.22) and Cluster (*p* = 0.45) configurations (Figure 2C).

### Total Distance

**Figure 3:**
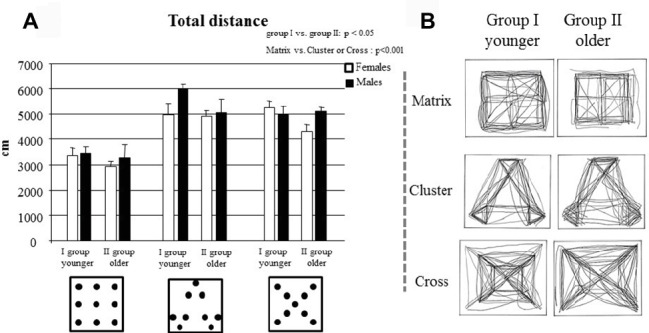
**(A)** Total distance of group I and group II travelled to complete the task in Matrix, Cluster, and Cross configurations. Data are presented as mean ± SEM. **(B)** Trajectories traveled by all children of each group are depicted.

A three-way ANOVA (Age × Gender × Configuration) revealed significant Age (*F*_1,32_ = 4.48, *p* = 0.04, ${\eta_{p}}^{2}$ = 0.12) and Configuration (*F*_2,64_ = 39.27, *p* < 0.000001, ${\eta_{p}}^{2}$ = 0.55) effects, while the Gender (*F*_1,32_ = 3.37, *p* = 0.08) effect was not significant. None of the interactions were significant (Age × Gender: *F*_1,32_ = 0.14, *p* = 0.71; Age × Configuration: *F*_2,64_ = 0.09, *p* = 0.91; Gender × Configuration: *F*_2,64_ = 0.29 *p* = 0.75; Age × Gender × Configuration: *F*_2,64_ = 2.20, *p* = 0.12). *Post hoc* comparisons performed on the Age effect showed that group I exhibited higher values of total distance than group I (*p* = 0.04). Moreover, *post hoc* comparisons performed on the Configuration effect showed that children exhibited higher values of total distance in the Cross and Cluster configurations than in the Matrix and Cluster configurations (Cluster or Cross vs. Matrix: at least *p* < 0.0001) (Figures 3A,B).

### Drawings

**Figure 4:**
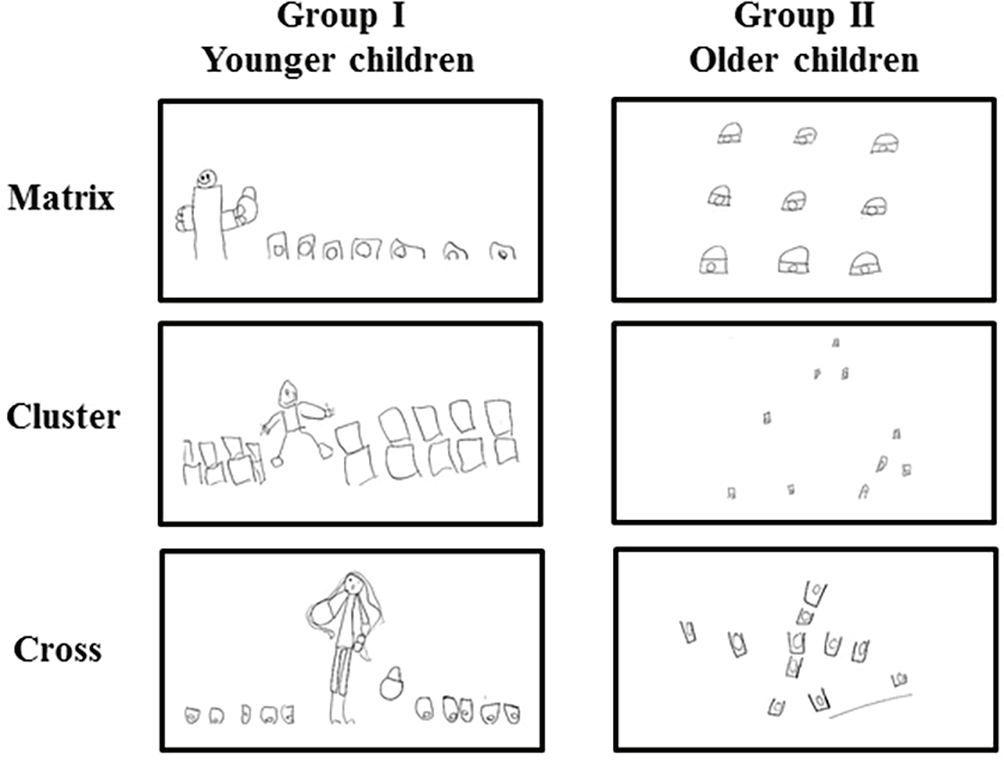
Selected drawings of group I and group II. At the end of each configuration, the children were required to draw the setting they had just experienced.

All children willingly drew the spatial setting where they had just “played.” A three-way ANOVA (Age × Gender × Configuration) revealed a significant Age effect (*F*_1,28_ = 6.55, *p* = 0.02, ${\eta_{p}}^{2}$ = 0.19), while Gender (*F*_1,28_ = 0.16, *p* = 0.69) and Configuration (*F*_2,56_ = 2.29, *p* = 0.11) effects were not significant. None of the interactions were significant (Age × Gender: *F*_1,28_ = 0.14, *p* = 0.71; Age × Configuration: *F*_2,56_ = 0.51, *p* = 0.6; Gender × Configuration: *F*_2,56_ = 0.89 *p* = 0.42; Age × Gender × Configuration: *F*_2,56_ = 1.72, *p* = 0.19). *Post hoc* comparisons performed on the Age effect revealed that the values of younger children (mean score: 1.24 ± 0.44) were significantly different in comparison to older children (mean score: 2.3 ± 0.81) (Figure 4).

## Discussion

The present research focused on the development of spatial abilities using ecological settings with different configurations and without spatial constraints. The three configurations children explored were placed outdoor. Thus, our experimental setting allowed children to consider themselves as participants in a search game, thus motivating them to perform the task. Another positive aspect of our task is that it allows the analyses of different facets of spatial memory. In fact, the analysis of all the parameters provides information on procedural competences, on declarative knowledge, on the mental representation of the environment, and on spatial working memory abilities.

The main results of the present study are severalfold.

Firstly, the development of spatial abilities follows a precise developmental trend with a clear age-related improvement. In particular, younger children displayed worse performances as compared to the older ones with regard to the total time employed to complete Cluster and Cross configurations, in the number of total and re-visit errors, in search efficiency, in spatial span, and in distance travelled (Figures 1–3). However, older children did not always have error-free performance or maximum span value, suggesting that at 6 years of age such abilities are not fully developed. To be confirmed, such a hypothesis should be further tested with older children. These findings are in accordance with previous developmental psychological evidence showing that children younger than 7 years of age fail to resolve spatial behavioral tasks (Overman et al., 1996; Lehnung et al., 1998; Mandolesi et al., 2009; Foti et al., 2011). Moreover, our study is in accordance with Lehnung et al. (2003) who have shown that locomotion facilitates the acquisition of declarative knowledge in children under the age of 7 and with Boccia et al. (2017) who have shown that navigational training enhances allocentric spatial recall. Our results suggest that the acquisition of declarative knowledge is more effective if the children are allowed to move in the open space, without spatial constraints. According to this, the children belonging to group II (mean age: 5.8 ± 0.9), besides scoring higher in all parameters as compared to children of group I, drew the configurations mainly as observed from above, thus suggesting a growing capacity of mental representation (Figure 4). However, their mental representative mapping abilities are not fully developed, as evidenced by their drawings, where the representation of the configurations is not always complete and still flawed by elements of egocentric perspective. Conversely, the drawings of younger children were characterized exclusively by the egocentric perspective. These data suggest a clear age-related improvement in the mental representative mapping abilities and support the idea that exploring the space appropriately is a necessary condition in order to build a cognitive spatial map (Mandolesi et al., 2003; Foti et al., 2018).

Other evidence provided in this paper concerns gender differences in solving the multiple reward task. We observed better performance of males than females in search efficiency and in no-visit errors in all configurations (Figures 1, 2) and better performance of males than females in total error and in the spatial span only for the Cross configuration (Figures 1, 2). As will be discussed later, the Cross configuration is the hardest to explore, and it is interesting to note that in this specific experimental condition gender differences emerged. Altogether, these data might appear to be in contradiction with our previous results (Mandolesi et al., 2009). In fact, we found a precocious acquisition of spatial competencies in females both in the procedural components and in the working memory abilities. However, it is important to stress that gender differences may vary widely depending on several factors, such as the spatial task used. In our previous work, we analyzed spatial abilities in children using the radial maze task that is strongly influenced by spatial constraints. Here, children have to explore an open space, without any spatial constraints, and therefore, they had to organize (plan) a path suitable for the configuration to be explored. Thus, it is reasonable to conclude that any gender difference observed in children in a given spatial task cannot be generalized to other spatial tasks. In particular, the Cross configuration is the hardest configuration, where the optimal strategy is not immediately suggested by the geometry. This feature requires further cognitive abilities, such as cognitive flexibility. In fact, the child has to change of strategy when finishing one line and starting a new one. Such peculiarity might make gender differences emerge in this specific task. Thus, one might speculate that spatial constraints are dealt with differently according to the gender of the participant. However, more studies will be needed to confirm or falsify such a hypothesis.

One more piece of evidence provided in this manuscript is that the environment strongly affects spatial exploration. In fact, as explained before, we observed that some configurations are easier to explore than others. In particular, in the Matrix and Cluster configurations, children made fewer re-visit errors and exhibited higher levels of search efficiency than they did in the Cross configuration (Figure 1). Moreover, the Matrix configuration was explored by traveling the shortest distance (Figure 3). To explain these differences, it is important to take into account the characteristics of the three configurations.

Efficient strategies for exploring the Matrix configuration are structured search patterns that follow rows (or columns) sequentially or, conversely, that travel the perimeter of the external “square” to reach the most internal bucket at the end (Foti et al., 2011, 2015). In previous studies, we highlighted that pre-school children explored the Matrix configuration using a structured search patterns characterized by the shortest transitions from one bucket to another (Foti et al., 2011, 2015), thus suggesting that children can orientate themselves in an open environment already at about 6 years of age, as long as structured internal patterns are present. This may be the reason why children explored the Matrix configuration more easily. The Cluster configuration offers the possibility of using a chunking strategy, first visiting the locations within the same cluster and then moving to another one. The chunking theory (Murdock, 1995, 2005; Schyns et al., 1998) predicts that, once the chunks have been retrieved, the burden on memory will be a function of the number of clusters to be explored in the search space (in our case, three) rather than of the total number of locations to be explored (in our case, nine). Thus, the chunking strategy implies a hierarchical organization of memory, substantially reducing the working memory load, thus improving the overall performance (Terrace and McGonigle, 1994; Cohen et al., 2003). Given its reduced mnesic load, even this configuration is not particularly difficult to explore. Hence, one might speculate that the hierarchical organization of particular facets of spatial memory starts to develop earlier than 4 years of age. Finally, the Cross configuration is characterized by strong spatial constraints. As explained before, the most effective strategy to fully explore the Cross configuration requires that the children use an end-to-end search pattern twice, moving along the lines and visiting the next bucket at each step. However, once a line is completed and the children reach its end, it is necessary to switch to the second line by reaching to the farthest bucket (thus modifying the strategy). This change of strategy requires cognitive flexibility, an ability that matures later on during the growth, along with the maturation of the frontal lobes (Oyefiade et al., 2018). This interpretation would explain why the Cross configuration is more difficult to explore in comparison to the Matrix and the Cluster ones.

In conclusion, it can be proposed that both the procedural competences and the memory load requested to explore a specific environment are determined by its specific features. The memory load required might partly explain the difficulties in the exploration of more complex environments by younger children who have not yet completed the maturation of cerebral areas involved in the processing of spatial memory. Likewise, the complexity of the environment to be explored requires specific spatial abilities, which might be related to the emergence of gender differences. Finally, our study shows how the exploration of the environment facilitates the building of its internal representation and highlights that movement plays an important role in the development of spatial abilities.

Overall, our findings provide information about the timing of the development of spatial orientation and spatial memory and are in line with previous evidence. Further investigation is needed to characterize the developmental trend of spatial cognitive functions.

## Author Contributions

All authors designed the research. AL and MP tested the participants. PS analyzed the data. All authors discussed the data. LM, FF, PS and GS wrote the paper. All authors read, revised, and approved the final manuscript.

### Conflict of Interest Statement

The authors declare that the research was conducted in the absence of any commercial or financial relationships that could be construed as a potential conflict of interest.
